# Corrosion Behavior of the As-Cast and As-Solid Solution Mg-Al-Ge Alloy

**DOI:** 10.3390/ma11101812

**Published:** 2018-09-24

**Authors:** Xiaoda Liu, Ming Yin, Shaohua Zhang, Huan Wei, Baosheng Liu, Huayun Du, Lifeng Hou, Yinghui Wei

**Affiliations:** 1 College of Materials Science and Engineering, Taiyuan University of Technology, Taiyuan 030024, China; liuxd@tyut.edu.cn (X.L.); ymym6868@163.com (M.Y.); zaqcde9850@163.com (S.Z.); wh201702@126.com (H.W.); duhuayun@tyut.edu.cn (H.D.); 2 College of Materials Science and Engineering, Taiyuan University of Science and Technology, Taiyuan 030024, China; liubaosheng@tyust.edu.cn

**Keywords:** Mg-3Al-*x*Ge alloy, second phase, corrosion, solid solution treatment, microstructure

## Abstract

The corrosion behavior of Mg-3Al-*x*Ge (*x* = 1, 3, 5) alloy in as-cast and as-solid was investigated by virtue of microstructure, corrosion morphology observation, and electrochemical measurement. Among the as-cast alloys, the corrosion rate of Mg-3Al-1Ge with a discontinuous bar-morphology was the highest, which was 101.7 mm·a^−1^; the corrosion rate of Mg-3Al-3Ge with a continuous network distribution was the lowest, which was 23.1 mm·a^−1^; and the corrosion rate of Mg-3Al-5Ge of Ge-enriched phase with sporadic distribution was in-between, which was 63.9 mm·a^−1^. It is suggested that the morphology of the Mg_2_Ge phase changes with a change in Ge content, which affects the corrosion performance of the alloy. After solid solution treatment, the corrosion rate of the corresponding solid solution alloy increased—Mg-3Al-1Ge to 140.5 mm·a^−1^, Mg-3Al-3Ge to 52.9 mm·a^−1^, and Mg-3Al-5Ge to 87.3 mm·a^−1^, respectively. After investigation of the microstructure, it can be suggested that solid solution treatment dissolves the Mg_17_Al_12_ phase, which changes the phase composition of the alloy and also affects its microstructure, thus affecting its corrosion performance.

## 1. Introduction

Magnesium alloy is a kind of metal material with low density and high specific strength. It is widely used in automotive, aerospace [1,2,3,4,5], medical [6,7,8], and other fields [9,10,11]; however, its poor corrosion resistance hinders its further development and application, meaning its corrosion behavior is an important research direction in the study of magnesium alloys.

Corrosion of magnesium alloys can be due to external factors such as environmental media, as well as internal factors such as composition and microstructure, especially the precipitated phase [12,13,14,15]. In regard to precipitation phases of magnesium alloy, it is generally believed that the precipitated phases can contribute to galvanic corrosion with the matrix, accelerating the corrosion of magnesium alloy. For example, Song and colleagues [16] reported that the accelerated corrosion of GW93 magnesium alloy can be attributed to the small amounts of precipitated phases. On the other hand, the corrosion resistance of magnesium alloy can be enhanced as a result of the reduction of the precipitated phases [17,18]. As such, the precipitated phases can be dissolved after solution treatment, which may be helpful in reducing the corrosion rate of magnesium alloy [19]. In contrast, the formation of network-precipitated phases blocks the corrosion of magnesium alloy and improves its corrosion resistance [20].

Mg-Al alloys are the earliest and most widely used magnesium alloys [21,22]. Adding alloying elements can improve the corrosion resistance of Mg-Al alloy by changing the microstructure and the distribution of the second phase, such as AZ91D, AM50 [23,24,25]. Recently, it has been found that the addition of Ge to binary alloy can effectively improve corrosion resistance. Kim [26] reported that the corrosion resistance of Mg-*x*Ge (*x* = 0.5~2 wt%) binary alloy can be effectively increased with the addition of the Ge element compared with that of pure magnesium, and implied that adding the Ge element did play a significant role. Liu [27] studied the electrochemical performance of Mg-*x*Ge (*x* = 0.1, 0.3) binary alloys and found that the addition of Ge could suppress corrosion ability though inhibiting the hydrogen evolution process.

With respect to Ge-addition ternary alloys, Liu [28] reported that the addition of Ge to Mg-Zn alloy can improve the corrosion resistance of the alloy, and the severe filiform corrosion morphology was changed into discrete surface corrosion. In contrast, Mg-Al-Ge ternary alloy is a potential structural material [29]; therefore, Islanm [30] studied the thermodynamic model of Mg-Al-Ge ternary alloys. However, the corrosion behavior of Mg-Al-Ge ternary alloy has hardly been reported in the literature. Therefore, it is of great significance to investigate the effect of Ge on the corrosion resistance of Mg-Al alloys.

The main objective of this work is to investigate the effect of Ge on the corrosion properties in various second relative Mg-Al-Ge ternary alloys in 3.5 wt% NaCl solution. Additionally, the contributing mechanism of the Ge alloying element will be clarified, and the influence of solid solution treated on the corrosion behavior of Mg-Al-Ge ternary alloys in 3.5 wt% NaCl solution was illustrated. As such, the ternary Mg alloys with different kinds of Ge content, such as Mg-3Al-1Ge, Mg-3Al-3Ge, and Mg-3Al-5Ge, were designed and smelted together with the corresponding solution treatments in the present study.

## 2. Experiment

### 2.1. Material Preparation

The experimental material used for this investigation were Mg-3Al-*x*Ge (*x* = 1, 3, 5) alloys. Based on the Mg-Al-Ge phase diagram [31], high-purity Magnesium, Aluminum, and Germanium (purity, 99.99 wt%) were melted in an induction melting furnace under the protection of a SF_6_ + CO_2_ atmosphere. Pure magnesium in the form of foil was used to pack Mg, Al, and Ge pieces to decrease their volatilization loss during the melting process. Mass losses of the alloys after melting were less than 1 wt%. The real chemical compositions of the alloys were determined by ICP-AES (Inductively Coupled Plasma-Atomic Emission Spectrometry) (Thermo Fisher iCAP6300, Shanghai, China). The chemical composition of Mg-Al-xGe alloys is listed in Table 1. The obtained Mg-3Al-xGe as-cast ingot was by solid solution treatment at 420 °C for 24 h, followed by quenching in cold water. Finally, a total of six Mg-Al-*x*Ge alloy samples were obtained, including three as-cast samples and three solid-solution samples, abbreviated as AG31, AG33, AG35, AG31H, AG33H, and AG35H, respectively.

### 2.2. Microstructure Characterization

The phase composition of Mg-3Al-xGe alloys were detected using XRD (TD-3500, Dandong Tongda Science and Technology Co., Ltd., Dandong, China). The microstructure characteristics were detected using SEM (VEGA3, TESCAN Co., Ltd., Shanghai, China), equipped with EDS.

XRD measurements were done with Cu K_α_ radiation. The scan range of 2θ was from 20° to 80° with a scan step of 0.02°. The XRD pattern was analyzed with MDI Jade software (5.0).

### 2.3. Electrochemical Measurements

A SP-150 electrochemical workstation (SP-150, Bio-logic Science Instruments, Seyssinet-Pariset, French) was used for the electrochemical measurements. A three-electrode electrolyte cell was utilized, which had a platinum plate as a counter electrode and a saturated calomel electrode (SCE) as a reference electrode. The working electrode was a Mg-3Al-*x*Ge alloy. Before the tests, the working electrode was immersed into the test solutions for 20 min until a steady-state open circuit potential (OCP) was established. Then, potentiodynamic polarization tests were carried out. The polarization curves were recorded at a scanning rate of 1 mV·s^−1^.

### 2.4. Immersion and Weight-Loss Measurements

Immersion tests were performed in 3.5 wt% NaCl solution for 24 h. After immersion, the specimens were cleaned to remove surface corrosion products, abiding by the ASTM G1 standard. Three parallel specimens before and after immersion were weighed with an electronic auto-balance (with 0.1 mg accuracy) to measure the average corrosion rates.

The corrosion rate *ν* (mm·a^−1^) measured by weighting loss can be calculated by the following equation:

(1) $$x=\frac{\left(w_{1}-w_{2}\right)\times 87,600}{A\times T\times D}$$

where *w*_1_ and *w*_2_ are the weight of specimens before and after immersion respectively; 87,600 is the calculation constant; *T* is immersion time; *A* is the total exposed surface area of specimens; and *D* is the density of specimens.

The immersion tests and electrochemical measurements were carried out at 25 ± 2 °C.

## 3. Results

### 3.1. Microstructure of Mg-3Al-xGe Alloy

**Figure 1:**
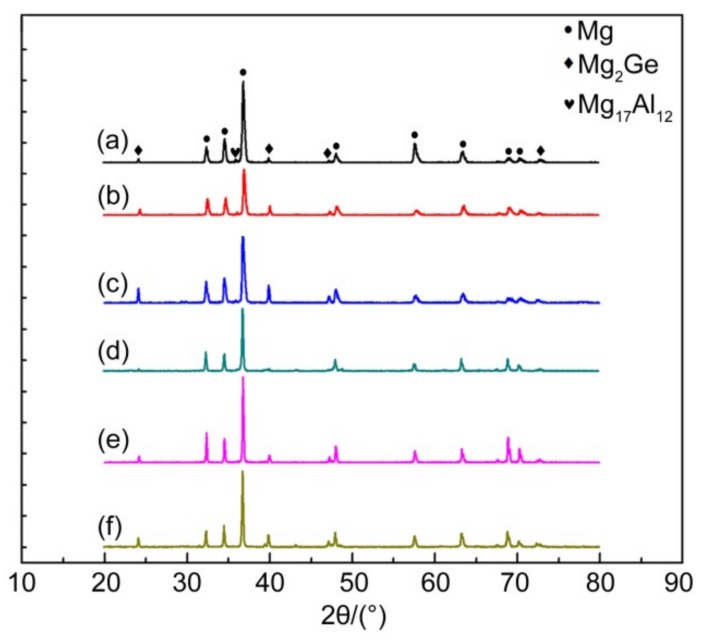
XRD pattern of Mg-3Al-*x*Ge alloy. (a) AG31, (b) AG33, (c) AG35, (d) AG31H, (e) AG33H, and (f) AG35H.

Figure 1 shows an XRD spectrum of the as-cast and solid-solution Mg-3Al-xGe alloy. The as-cast alloys contain the β-Mg_17_Al_12_ phase, Mg_2_Ge phase, and α-Mg matrix. Interestingly, the peak of the Mg_2_Ge-phase enhancement was with the increase in Ge content, while the peak of the β-Mg_17_Al_12_ phase is weaker, indicating that the Mg_2_Ge-phase precipitates gradually increase and the β-Mg_17_Al_12_ phase precipitates less. The microstructure of the as-solid solution alloy contains the Mg_2_Ge phase and α-Mg matrix, while the β-Mg_17_Al_12_ phase disappears after solution treatment. In contrast, it is difficult for the Mg_2_Ge phase to dissolve into the Mg matrix after solid-solution treatment because the solubility of Ge in Mg is low [32], so the change in the peak of Mg_2_Ge is consistent with that of the as-cast alloy.

**Figure 2:**
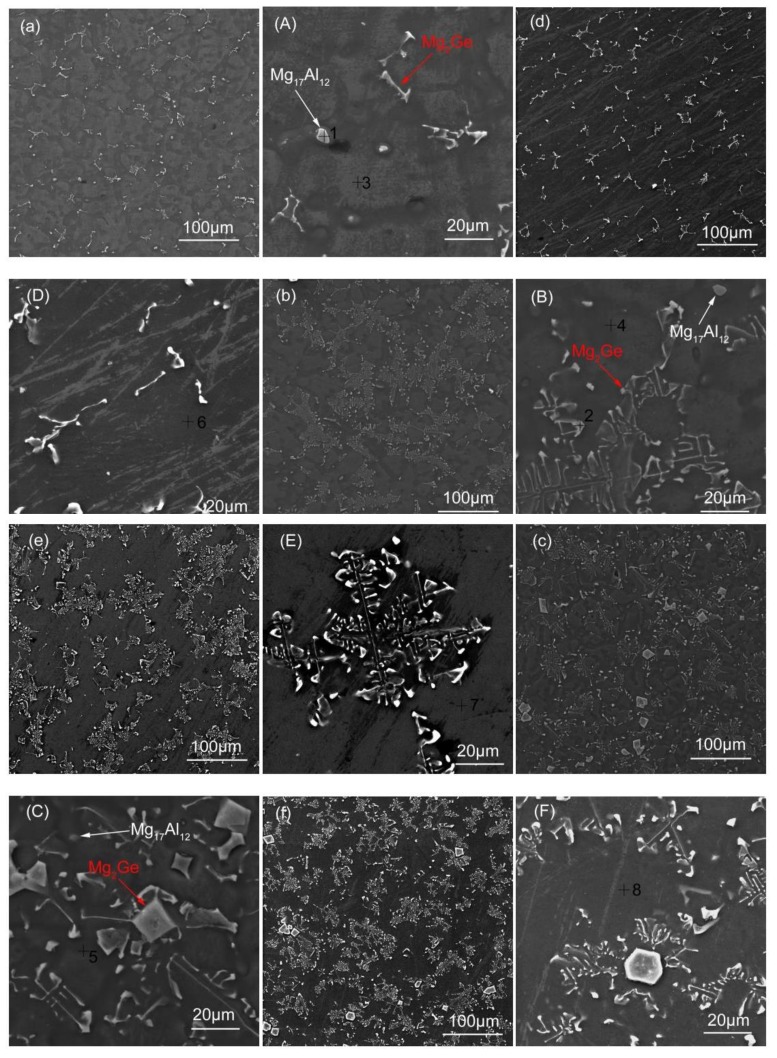
Microstructure of Mg-3Al-*x*Ge alloy. (**a**) AG31, (**A**) high magnification AG31, (**b**) AG33, (**B**) high magnification AG33, (**c**) AG35, (**C**) high magnification AG35, (**d**) AG31H, (**D**) high magnification AG31H, (**e**) AG33H, (**E**) high magnification AG33H, (**f**) AG35H, and (**F**) high magnification AG35H.

Figure 2 shows the microstructure of the as-cast and solid solution Mg-3Al-*x*Ge alloy. In the case of the high magnification morphology of as-cast Mg-3Al-*x*Ge alloys, as shown in Figure 2b,d,f, there are two kinds of precipitation phases can be observed, Mg_17_Al_12_ and Mg_2_Ge, and they are marked with the white arrow and red arrow, respectively. With respect to the solid-solution Mg-3Al-*x*Ge alloys, a significant change in microstructure can be seen. There are still plenty of second-phase Mg_2_Ge observed on the grain boundaries, but less of those of the β-Mg_17_Al_12_ phase (Figure 2h,j,l), indicating the complete dissolution of the β-Mg_17_Al_12_ phase into the α-Mg matrix. In general, no matter whether they are as-cast or solid-solution alloys, the Mg_2_Ge phase presents a discontinuous and inhomogeneous distribution for the bar-morphology in the AG31 (as shown in Figure 2a,g), a continuous and homogeneous distribution in the form of a network in the AG33 (as shown in Figure 2c,i), and a block and inhomogeneous distribution with a little bit of Mg_2_Ge second-phases gathering in the AG35 alloy on the basis of network shape (as shown in Figure 2e,k). The microstructure of Mg-3Al-*x*Ge alloy is in good agreement with the XRD results. The element composition of points 1–8 in Figure 2 measured by EDX (Energy Dispersive X-Ray Spectroscopy) is presented in Table 2, and the element composition of point 1 is composed of Mg and Al, indicating that the white precipitation particles are Mg_17_Al_12_. The element composition of point 2 is composed of Mg and Ge, indicating that the white long-bar precipitation phase is Mg_2_Ge. The element composition of Points 3,4,5 are composed of Mg, Al, and Ge. In addition, the element composition of Points 6,7,8 are composed of Mg and Al, indicating that the matrix of as-cast alloys have Ge, and there is no Ge after solid-solution treatment.

### 3.2. Electrochemical Test

**Figure 3:**
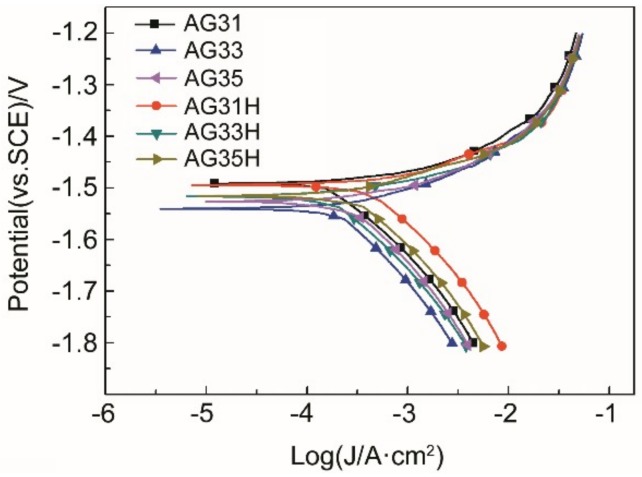
Polarization curves of as-cast and solid-solution Mg-3Al-xGe alloys at 25 °C.

Figure 3 shows the polarization curves of the as-cast and solid-solution Mg-3Al-*x*Ge alloy in 3.5 wt% NaCl solution. It can be seen from the Tafel curve that the corrosion current density (icorr) in the anode region of the polarization curve is basically the same, and the icorr in the cathode region changes greatly, indicating that the corrosion processes are controlled by the hydrogen evaluation reaction of cathodic sides [33]. Besides, the anodic current density increased steadily with the applied potential for all specimens, indicating the active dissolution of Mg. None of the specimens exhibited a passive region. The fitting results of the polarization curves are listed in Table 3. According to Table 3, the icorr values of the solid-solution Mg-3Al-*x*Ge alloys is higher than that of corresponding as-cast specimens, which demonstrates that the as-cast Mg-3Al-*x*Ge alloy exhibits a lower corrosion rate than solid-solution Mg-3Al-*x*Ge alloy. The icorr values of the as-cast and solid-solution Mg-3Al-*x*Ge alloys have the same changing rule; that is, the icorr of AG33 alloy is the smallest, that of AG31 is the largest, and that of AG35 is in-between. This indicates that the corrosion rate of AG33 is the lowest and the corrosion rate of AG31 is the highest. However, the corrosion potentials (Ecorr) of these alloys are concentrated within a small range, and the Ecorr gradually becomes negative as the Ge content increases.

### 3.3. Immersion and Mass-Loss Test

**Figure 4:**
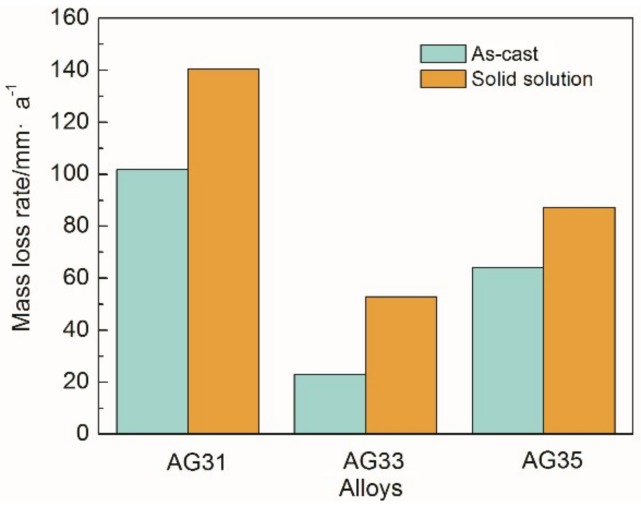
Mass-loss rate of the as-cast and solid-solution Mg-3Al-*x*Ge alloys in 3.5% NaCl solution for 18 h.

Moreover, the corrosion resistance of Mg-3Al-*x*Ge alloys was investigated by immersion tests in 3.5% NaCl solution for 18 h. Figure 4 shows the mass loss rate of as-cast and solid-solution Mg-3Al-*x*Ge alloys. It was found that the mass loss rate of the three as-cast and solid solution specimens is in the order of Mg-3Al-3Ge (23.081 mm·a^−1^ and 52.889 mm·a^−1^) < Mg-3Al-5Ge (63.974 mm·a^−1^ and 87.298 mm·a^−1^) < Mg-3Al-1Ge (101.734 mm·a^−1^ and 140.524 mm·a^−1^), indicating a decrease in corrosion resistance in the same order. Similarly, as-cast Mg-3Al-*x*Ge alloys present better corrosion resistance than that of corresponding solid-solution specimens. The mass-loss rate results are in good agreement with the polarization curves and hydrogen evolution data. The above results imaged that the alloy with alloying element content have discriminating corrosion resistance, ascribed to the precipitations of the second phase.

**Figure 5:**
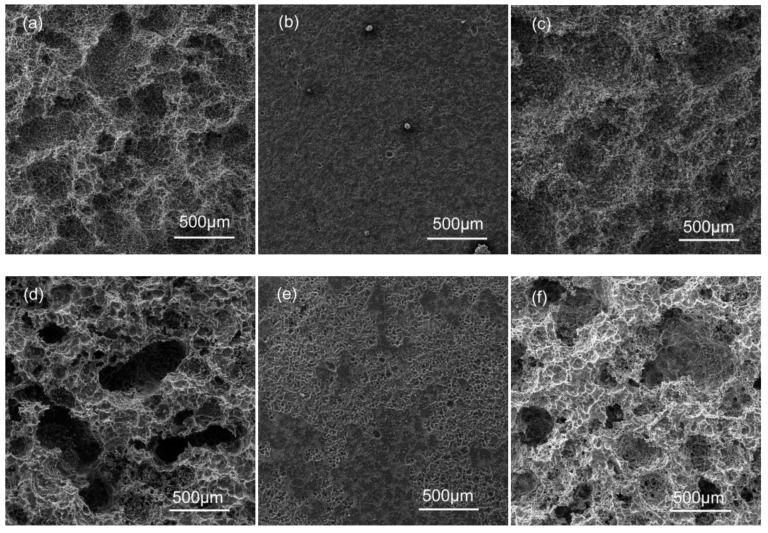
SEM morphologies of the sample surfaces after removing corrosion products: (**a**) AG31, (**b**) AG33, (**c**) AG35, (**d**) AG31H, (**e**) AG33H, and (f) AG35H.

Figure 5 shows SEM images of the sample surfaces after removing the corrosion products after conducting immersion experiments in 3.5% NaCl for 18 h. All specimens were susceptible to pitting corrosion. In the as-cast alloys, a large number of small shallow corrosion pits appear in the AG31 alloy (Figure 5a), and the small corrosion pit gradually expands into a large, deep corrosion pit. The AG33 alloy (Figure 5b) has many small corrosion pits that are evenly distributed, and no large corrosion pits appear. The AG35 alloy (Figure 5c) also has many small corrosion pits, but the large corrosion pits are shallower than the AG31 alloy. In the as-solid solution alloys, the small corrosion pit of the AG31H (Figure 5d) alloy is significantly larger than the small corrosion pit size of the as-cast AG31 alloy, and large and deep corrosion pits with uneven distribution appear. The AG33H alloy (Figure 5e) has many small corrosion pits that are unevenly distributed and has a greater corrosion depth than the as-cast AG33 alloy. On the basis of small corrosion pits, the AG35H alloy (Figure 5f) has large and deep corrosion pits that are unevenly distributed, which is deeper than the as-cast AG35, but not deeper than AG31H. As shown in Figure 5, the corrosion rate of the as-cast alloys were lower than that of the corresponding as-solid solution alloys, and the corrosion rate of alloys with different Ge content were AG31, AG35, and AG33, respectively.

## 4. Discussion

The main factors that affected the corrosion of magnesium alloys by the precipitation phase are galvanic corrosion [13], mechanical blocking corrosion [15], and uniform corrosion current density [33]. According to the microstructure, electrochemical test and immersion test, it can be found that there are two factors affecting the corrosion of Mg-3Al-*x*Ge alloys.

### 4.1. Effect of Ge Content on Corrosion Behavior of Mg-3Al-xGe Alloys

According to the results of Figure 4 and Figure 5, it can be found that the corrosion rate of AG31 is the highest, that of AG33 is the lowest, and that of AG35 is in-between, both in as-cast or in as-solid solution alloys. In combination with the morphology of Figure 2, it is considered that the Mg_2_Ge phase is a rod-like precipitation in the AG31 alloy, which is dispersed and unevenly distributed. Therefore, the Mg_2_Ge phase will have intense galvanic corrosion with the matrix, which increases the corrosion current density and thus causes the corrosion rate to increase. The continuous and homogenization distribution of second phases (a net shape) in the AG33 alloy is shown in Figure 2b,e. It should be noted that second-phase fraction is a decisive factor that determines its role, which is either galvanic-accelerating or anodic-blocking [34]. In this study, the gaps between Mg_2_Ge precipitates are narrow and the Mg_2_Ge phase is an almost continuous network. Due to the presence of large amounts of Mg_2_Ge phases in the AG33 alloy, the corrosion of the phase is quite easily obstructed by corrosion products on its surface, thus greatly delaying corrosion. Moreover, the Mg2Ge distributed through homogenization will increase the randomicity of galvanic corrosion, cause corrosion to occur uniformly, and also reduce the corrosion current density [33].

On the other hand, if the Mg_2_Ge phase is agglomerated and the distance between the Mg_2_Ge phases is large (as shown in Figure 2c,f), the Mg matrix cannot be effectively blocked by the Mg_2_Ge phase or by the corrosion products deposited between the second phases and the Mg matrix. As such, accelerated corrosion due to micro-galvanic corrosion is observed in the AG35 alloy.

### 4.2. Effect of Solid Solution Treatment on Corrosion Behavior of Mg-3Al-xGe Alloys

Solid-solution treatment can change the corrosion rate of the Mg-3Al-*x*Ge alloy by changing the microstructure of the alloy.

It is common for galvanic corrosion of magnesium alloys to be reduced and for corrosion resistance to improve as a result of the complete dissolution of second phases after solid-solution treatment [35]. In Mg-Al-Ge alloys, the *β* phase (Mg_17_Al_12_) dissolves into an Mg matrix, as shown in Figure 2D–F. In contrast, the Mg_2_Ge phase does not dissolve after the corresponding solid-solution treatment due to its low solubility in Mg [32]. It should be noted that, as shown in Table 2, the Ge element in the Mg matrix disappears after the solid-solution treatment, which reveals the possible re-precipitation of small amounts of Mg_2_Ge phases during the dissolution process of the *β* phase. The remaining Mg_2_Ge phase can reduce the corrosion resistance of magnesium alloys to a great degree.

Additionally, the potential difference in the galvanic coupling of Mg and Al can initially partly hinder the surface thermodynamic refining actions, accelerate the diffusion of the aggressive Cl^−^ ions in the electrolyte solution to the active corrosion sites on Mg matrix, and consequently speed up its corrosion [36].

As such, the accelerated corrosion of magnesium alloys proceeds despite the removal of the *β* phase after solid-solution treatment.

## 5. Conclusions

There are two precipitates in the as-cast Mg-3Al-*x*Ge alloy—Mg_17_Al_12_ and Mg_2_Ge—and there are trace Ge elements in the matrix. After solid solution, only the Mg_2_Ge phase is found in the Mg-3Al-*x*Ge alloy, the Mg_17_Al_12_ phase is dissolved, and there is no Ge element in the matrix.

As the content of Ge increases, the precipitation morphology of the Mg_2_Ge phase changes. In the Mg-3Al-1Ge alloy, the Mg_2_Ge phase is a dispersed, non-uniformly distributed, rod-like precipitated phase. In the Mg-3Al-3Ge alloy, the Mg_2_Ge phase is a uniform distribution of the network precipitates. In the Mg-3Al-5Ge alloy, a bulk Mg_2_Ge phase appears, which is sporadic in the network precipitate.

In the as-cast and as-solid solution Mg-3Al-*x*Ge alloys, the corrosion rate from small to large is followed by AG33, AG35, and AG31. The corrosion rate of the as-solid solution alloy is higher than that of the corresponding as-cast alloy. All Mg-3Al-*x*Ge alloys were susceptible to pitting corrosion and did not show a passivation region.

## Figures and Tables

**Table 1:** Chemical compositions of Mg-Al-Ge alloy in this experiment (wt%).

Samples	Al	Ge	Si	Mn	Fe	Mg
AG31	3.15	0.89	0.042	0.015	0.018	Bal.
AG33	3.05	3.14	0.045	0.013	0.016	Bal.
AG35	3.20	4.85	0.043	0.014	0.017	Bal.

**Table 2:** Element composition of the points marked as 1, 2, 3, 4, 5, and 6 in Figure 2.

Point	Mg		Al		Ge	
	wt%	at%	wt%	at%	wt%	at%
1	67.09	69.37	32.91	30.63	-	-
2	72.53	88.75	-	-	27.47	11.25
3	97.07	97.78	2.45	2.22	0.48	0.16
4	97.01	97.63	2.38	2.16	0.61	0.21
5	96.76	97.52	2.42	2.20	0.82	0.28
6	96.39	96.74	3.61	3.26	-	-
7	96.24	96.60	3.76	3.40	-	-
8	96.58	96.91	3.42	3.09	-	-

**Table 3:** Electrochemical parameters of the polarization curves of as-cast and solid-solution Mg-3Al-*x*Ge alloys.

Alloy	*E_corr_* (V vs. SCE)	*i_corr_* (μA/cm^2^)	*β*_a_ (V/dec)	*β*_c_ (V/dec)
AG31	−1.49	518.7	93.8	505.3
AG33	−1.52	292.7	91.2	339.4
AG35	−1.54	455.7	102.5	256.4
AG31H	−1.49	1 166.7	119.5	596.3
AG33H	−1.52	312.7	73.0	236.5
AG35H	−1.53	745.8	113.2	432.3
